# Determination of Retinol, Cholecalciferol, α-Tocopherol and Phylloquinone Levels in Dogs with Dilated Cardiomyopathy: A Preliminary Study

**DOI:** 10.3390/ani15172477

**Published:** 2025-08-23

**Authors:** Bengü Bilgiç, Muhammed Işık, Ahmet Bakır, Suat Ekin, Süleyman Kozat, Michela Pugliese, Mehmet Erman Or

**Affiliations:** 1 Department of Internal Medicine, Faculty of Veterinary Medicine, Istanbul University-Cerrahpasa, 34320 Istanbul, Turkey; ermanor@iuc.edu.tr; 2 Department of Internal Medicine, Faculty of Veterinary Medicine, Van Yuzuncu Yil University, 65100 Van, Turkey; muhammedisik@yyu.edu.tr (M.I.); skozat@yyu.edu.tr (S.K.); 3 Department of Chemistry, Division of Biochemistry, Faculty of Science, Van Yuzuncu Yil University, 65080 Van, Turkey; ahmetbakir@yyu.edu.tr (A.B.); suatekin@yyu.edu.tr (S.E.); 4 Department of Veterinary Sciences, University of Messina, 98168 Messina, Italy; michela.pugliese@unime.it

**Keywords:** dog, DCM, fat-soluble vitamins, heart

## Abstract

Lipid-soluble vitamins are essential micronutrients that play a vital role in processes such as growth, reproduction, immune regulation, antioxidant defense, anti-inflammatory response and cardiovascular health. This study aimed to evaluate the plasma levels of retinol, cholecalciferol, α-tocopherol and phylloquinone in dogs with dilated cardiomyopathy (DCM). Six dogs diagnosed with DCM and ten healthy control dogs were selected based on clinical, echocardiographic, electrocardiographic and radiographic examinations. Chromatographic analysis was performed to analyze the content of lipid-soluble vitamins. The mean plasma retinol levels were 0.0285 μg/mL and 0.0495 μg/mL in the DCM and control groups, respectively, and the mean cholecalciferol levels were 0.0739 μg/mL and 0.1045 μg/mL in the DCM and control groups, respectively. Retinol and cholecalciferol levels were both statistically lower in the DCM group than in the control group (*p* < 0.05). The mean plasma α-tocopherol levels were 0.3103 and 0.4322 μg/mL, and the mean plasma phylloquinone levels were 0.03780 and 0.04082 μg/mL in the DCM and control groups, respectively. However, no statistical significance was observed between the two groups for α-tocopherol or phylloquinone levels. Based on our findings, alterations in vitamin A and D metabolism could be associated with the development of idiopathic DCM in dogs. However, given the limited sample size, further investigation in larger populations is needed.

## Simple Summary

Lipid-soluble vitamins are essential micronutrients that play a vital role in processes such as growth, reproduction, immune regulation, antioxidant defense, the anti-inflammatory response and cardiovascular health. This study aimed to evaluate plasma levels of retinol, cholecalciferol, α-tocopherol and phylloquinone in dogs with dilated cardiomyopathy (DCM). Six dogs diagnosed with DCM and ten healthy controls were included in this study. Chromatographic analysis was performed to analyze the content of lipid-soluble vitamins. Retinol and cholecalciferol levels were both statistically lower in the DCM group than in the control group (*p* < 0.05). No statistical significance was observed between the two groups for α-tocopherol and phylloquinone levels. Based on these results, we recommend considering vitamin A and D supplementation alongside the standard treatment protocol for dogs diagnosed with DCM.

## 1. Introduction

Dilated cardiomyopathy (DCM) is a primary myocardial disease characterized by significant enlargement of the left ventricle or both ventricles and impaired systolic function. This can result in congestive heart failure, arrhythmias or sudden cardiac death [1]. It is one of the most commonly diagnosed acquired heart diseases in dogs, representing a significant cause of morbidity and mortality, particularly among large and giant breeds. Affected breeds include the Doberman Pinscher, Boxer, Great Dane, German Shepherd, Golden Retriever, Labrador Retriever, Rottweiler, Cocker Spaniel, Irish Wolfhound and Newfoundland [2,3]. Although DCM is often diagnosed as idiopathic, various factors have been associated with its development. These include genetic mutations affecting structural proteins, abnormalities in energy metabolism, viral myocarditis, autoimmune mechanisms, endocrine disorders and exposure to cardiotoxic drugs such as doxorubicin and NSAIDs [4,5,6,7].

In recent years, increasing attention has been paid to the potential role of nutrition in the development of DCM. Several case reports and observational studies have described diet-associated DCM in dogs consuming grain-free diets, particularly those high in legumes such as peas, lentils and chickpeas [8,9,10]. Some of these diets may be deficient in taurine, carnitine and other essential nutrients necessary for myocardial function, particularly in genetically predisposed breeds. Studies have shown that dietary intervention and supplementation with taurine or L-carnitine can lead to partial or complete clinical and echocardiographic recovery in some dogs, which further supports the link between diet and cardiac dysfunction. However, the precise mechanisms by which these dietary deficiencies contribute to myocardial injury are still under investigation [11,12].

Alongside these developments in veterinary medicine, research in human cardiology has increasingly focused on the role of micronutrients, particularly fat-soluble vitamins, in cardiovascular health and disease. For example, cholecalciferol (vitamin D_3_) has been identified as a key modulator of cardiovascular function. It acts via vitamin D receptors (VDRs), which are steroid hormone receptors found in cardiomyocytes, endothelial cells and vascular smooth muscle cells [13]. Deficiency in vitamin D has been associated with systemic inflammation, hypertension, endothelial dysfunction, left ventricular hypertrophy and an increased risk of heart failure in humans [14,15]. Clinical studies have shown that vitamin D_3_ supplementation downregulates the renin–angiotensin–aldosterone system (RAAS), reduces oxidative stress and improves myocardial contractility [15,16,17]. Another fat-soluble vitamin with potential cardioprotective effects is vitamin E, which includes tocopherols and tocotrienols in α, β, γ and δ forms. α-Tocopherol, the most biologically active form, is a potent antioxidant which protects lipid membranes from peroxidation and maintains the integrity of cardiomyocyte membranes under conditions of oxidative stress [18]. Several human studies have reported that adequate α-tocopherol intake is associated with reduced oxidative damage, decreased inflammation, and a lower risk of atherosclerosis and ischemic heart disease [16]. Retinol (vitamin A) is another essential fat-soluble vitamin, known for its roles in vision, epithelial cell maintenance and immune modulation. However, emerging evidence has highlighted its significance in cardiovascular biology. Retinol and its active metabolite, all-trans retinoic acid (ATRA), play a critical role in early embryonic heart development, myocardial regeneration and the preservation of left ventricular structure and function [19]. Experimental studies have demonstrated that retinoid signaling regulates cardiomyocyte differentiation and may mitigate ventricular remodeling following myocardial injury [20]. Retinol also influences the renin–angiotensin system and exhibits anti-inflammatory properties; both of these factors are relevant to the pathophysiology of heart failure [21]. Phylloquinone (vitamin K_1_), which is obtained primarily from green leafy vegetables such as collards, spinach, broccoli, cabbage and iceberg lettuce [22] and is supplemented in commercial diets, plays a pivotal role in blood coagulation by activating vitamin K-dependent proteins. However, recent data suggest that it may also contribute to cardiovascular homeostasis by regulating vascular calcification [23]. The Matrix Gla protein (MGP), a vitamin K-dependent inhibitor of vascular mineralization, requires adequate vitamin K for activation. Studies in human populations have associated higher dietary intake of phylloquinone with reduced coronary artery calcification and lower cardiovascular risk, even in younger individuals and adolescents [24,25].

Despite extensive research into the cardiovascular effects of these vitamins in humans, similar studies in veterinary medicine are scarce. In canine cardiology, for example, the impact of micronutrient levels on the development, progression or outcome of dilated cardiomyopathy (DCM) is not yet fully understood. However, given the known biological roles of vitamins A, D, E and K in processes such as cardiac metabolism, modulation of oxidative stress, calcium homeostasis and gene expression, it is possible that they could play a significant role in maintaining myocardial health. Furthermore, as DCM may be influenced by genetic susceptibility, as well as environmental or nutritional factors, it is vital to explore these relationships in order to develop a holistic understanding of the disease and effective preventive or therapeutic strategies. We hypothesize that serum concentrations of the fat-soluble vitamins retinol, cholecalciferol, α-tocopherol and phylloquinone differ significantly between dogs with DCM and healthy controls, reflecting their potential involvement in the pathophysiology of the disease. The present study, therefore, aimed to investigate the serum concentrations of four fat-soluble vitamins in dogs diagnosed with DCM. By determining hematological and biochemical values of all animals and examining potential deficiencies or alterations in vitamin levels, this study aims to shed light on possible associations between micronutrient status and myocardial dysfunction.

## 2. Materials and Methods

### 2.1. Animals and Study Design

This was an observational case–control study conducted between 2022 and 2024. A total of sixteen dogs of various breeds, weights, sexes and ages were included in this study. Six dogs diagnosed with dilated cardiomyopathy (DCM) and ten clinically healthy control dogs were selected based on clinical, echocardiographic, electrocardiographic and radiographic examinations.

Two-dimensional echocardiographic measurements were taken to record the left atrial (LA) and aortic (Ao) diameters, as well as the LA/Ao ratio. M-mode measurements included systolic and diastolic interventricular septal thicknesses (IVSs and IVSd, respectively), systolic and diastolic left ventricular internal diameters (LVIDs and LVIDd), systolic and diastolic left ventricular wall thicknesses (LVWs and LVWd), fractional shortening (FS), ejection fraction (EF) and E-point septal separation (EPSS). These measurements were taken using a Mindray Vetus 8 (Mindray Bio-Medical Electronics Co., Ltd., Shenzhen, China). All echocardiographic measurements were performed by a single experienced operator to reduce inter-observer variability. ECG evaluation was performed using a CONTEC^®^ ECG600G model 6-channel ECG device (CONTEC^®^, Shijiazhuang, China). The major criteria for the DCM group were left ventricular systolic enlargement, an increased sphericity index (<1.65) and a decrease in fractional shortening (<20–25%) or ejection fraction (<40%). The minor criteria were arrhythmias, an increased EPSS (>0.77 cm), a fractional shortening between 20 and 25% or less than 30%, and left atrial or biatrial dilation. According to the diagnostic scoring system proposed by Dukes-McEwan et al. (2003) [26], each major criterion was assigned 3 points and each minor criterion 1 point. A total score of ≥6 points was considered highly suggestive of subclinical DCM.

In symptomatic dogs, clinical signs such as exercise intolerance, dyspnea and coughing were evaluated, along with radiographic indicators of cardiac remodeling—specifically, the vertebral heart score and chamber enlargement—and electrocardiographic findings from a 1 min screening ECG for arrhythmias. A routine complete blood count (ProCyte Dx^®^, IDEXX Laboratories, Inc., Westbrook, ME, USA), serum biochemistry and total T4 measurements (Catalyst One^®^, IDEXX Laboratories, Inc., Westbrook, ME, USA) were performed on all dogs to exclude concurrent systemic diseases (Table 1 and Table 2). Dogs without clinical signs or a history of hypothyroidism and with serum total T4 concentrations within the reference range of 1.3–2.9 µg/dL were included in this study.

To minimize the influence of dietary variability on the parameters measured in this study, strict inclusion criteria were applied to ensure uniform nutritional intake among all subjects. Specifically, all dogs enrolled in this study were fed the same standardized commercial dry food for at least three months prior to sample collection. This diet was selected to provide complete and balanced nutrition according to established guidelines (FEDIAF) and to eliminate any potential confounding effects associated with variability in nutrient composition. Furthermore, it was confirmed that none of the dogs had received any vitamin or mineral supplements during this period, thereby eliminating the risk of artificially elevated or suppressed serum vitamin concentrations due to supplementation. All dogs were otherwise clinically healthy apart from their cardiac condition and exhibited no evidence of systemic illnesses that could influence the parameters under investigation. The dogs diagnosed with congestive heart failure at the initial clinical examination were not included in this study.

To ensure consistency and minimize potential confounding factors, several exclusion criteria were applied. These included (i) administration of vitamin or mineral supplements within the past three months, as such supplementation could interfere directly with serum levels of fat-soluble vitamins and compromise data interpretation; (ii) consumption of non-standardized, unregulated, homemade or raw diets, including grain-free formulations, which have been associated with nutritional imbalances and variable bioavailability of micronutrients; (iii) the presence of concurrent systemic diseases, such as hepatic dysfunction, renal insufficiency and endocrine disorders (e.g., hypothyroidism, diabetes mellitus and hyperadrenocorticism) or neoplastic conditions, as determined by comprehensive clinical evaluation, hematology and biochemistry profiles, urinalysis and diagnostic imaging; and (iv) treatment with pharmacological agents known to affect cardiovascular, renal or metabolic function, including corticosteroids, diuretics, inotropes and anticonvulsants; (v) a known history or diagnosis of cardiac diseases other than dilated cardiomyopathy, such as myxomatous mitral valve disease, pericardial effusion, subaortic stenosis or atrial septal defect, which could confound cardiac-specific parameters; (vi) pregnancy or lactation, due to physiological changes in metabolism, hormone regulation and cardiovascular dynamics that may affect vitamin distribution and cardiac measurements; (vii) an age of under one year, to eliminate developmental variability in cardiac morphology, enzymatic function and vitamin metabolism that are characteristic of juvenile physiology; (viii) stray and shelter dogs.

These rigorous inclusion and exclusion criteria were implemented to establish a homogeneous population with respect to nutritional status, systemic health and baseline metabolic function. This approach was intended to improve the reliability and clarity of the findings concerning the relationship between serum vitamin levels and dilated cardiomyopathy in dogs.

### 2.2. Vitamin Analysis (A, D, E and K)

Approximately 3 mL of whole blood was collected from the jugular vein of each dog and transferred into serum separator tubes. The samples were centrifuged at 3000 rpm for 10 min at room temperature. The obtained 1.5 mL serum was transferred into Eppendorf tubes and stored at −20 °C in the dark until the day of analysis.

Stock solutions of α-tocopherol, retinol, phylloquinone and cholecalciferol were prepared at a concentration of 500 μg/mL. To prepare the standard solutions, the stock solutions were appropriately diluted with methanol. For calibration, a linear regression analysis of the peak areas was performed to standardize the solution concentrations.

#### 2.2.1. Extraction Process

To minimize sample degradation due to UV light exposure, the samples were thawed at ambient temperature under fluorescent lights, covered with plastic sleeves.

α-Tocopherol, retinol, phylloquinone and cholecalciferol in serum were extracted as follows: 100 μL of serum was deproteinized by adding 100 μL of ethanol (Merck KGaA, Darmstadt, Germany), along with antioxidants such as 0.025% BHT (Sigma-Aldrich, St. Louis, MO, USA), to the extraction solvent. The samples were vortexed for 1 min. The samples were extracted twice with 600 μL of n-hexane (Merck KGaA, Darmstadt, Germany). After vortexing, the samples were centrifuged at 8000 rpm for 10 min. A total of 500 μL of the hexane layer was extracted and evaporated to dryness under a nitrogen stream at 37 °C. The residue was dissolved in 50 μL of tetrahydrofuran (Sigma-Aldrich, St. Louis, MO, USA), which was then added to 150 μL of methanol. After vortexing for 1 min, 100 μL samples were autosampled using amber glass vials (Agilent Technologies®, Waldbronn, Germany).

#### 2.2.2. Chromatographic Conditions

High-performance liquid chromatographic analyses were performed using an HP 1100 Series system (Agilent Technologies^®^, Waldbronn, Germany) equipped with a G1328 diode array detector (DAD) and a G1329 ALS autosampler set at −8 °C. A 5 μm GL Sciences C18 reversed-phase column (250 × 4.6 mm ID) was used for separation (250 × 4.6 mm ID; GL Sciences Inc., Tokyo, Japan). The mobile phase, consisting of a methanol-tetrahydrofuran mixture (80:20, *v*/*v*), was then modified [27]. The pump was set to a flow rate of 1.5 mL/min. Chromatographic analysis was performed at 40 °C using isocratic elution. The chromatogram was monitored using DAD array detection at 290, 325, 265 and 248 nm to simultaneously measure α-tocopherol, retinol, cholecalciferol and phylloquinone, respectively.

### 2.3. Statistical Analysis

SPSS version 22.0 (SPSS Inc., Chicago, IL, USA) was used for statistical analysis. The results are expressed as the arithmetic mean ± standard error of the mean (M ± SEM). Data distribution was assessed using the Shapiro–Wilk test. For variables that did not meet the assumption of normality, group comparisons were conducted using the Mann–Whitney U test; for normally distributed variables, the independent samples *t*-test was performed. A *p*-value < 0.05 was considered statistically significant.

## 3. Results

Of the dogs in the DCM group, four were male Golden Retrievers, one was a female Cocker Spaniel and one was a female mixed-breed dog. The healthy group included three female Cavalier King Charles, one female and one male Maltese Terrier, one female Yorkshire Terrier, one male Spitz, one female Golden Retriever, one male and one female mixed breed. The mean body weight was 26.33 ± 10.78 for the DCM group and 10 ± 5.31 for the healthy group. The mean age was 11 ± 1.41 for the DCM group and 5.4 ± 2.1 for the healthy group. Complete blood count and serum biochemistry values were within the reference ranges for all dogs (see Table 1 and Table 2). The mean vertebral heart score was 11.2 in the DCM group and 10 in the healthy control group.

A total of six dogs diagnosed with dilated cardiomyopathy (DCM) and ten clinically healthy dogs underwent comprehensive transthoracic echocardiographic evaluation. In the DCM group, the left ventricular internal diameter in diastole (LVIDd) ranged from 3.95 to 7.83 cm. The mean values in this group significantly exceeded those observed in the healthy control group, where the mean LVIDd was 2.64 cm. Similarly, the left ventricular internal diameter in systole (LVIDs) ranged from 3.07 to 4.78 cm in the affected dogs, which was markedly higher than the mean value of 1.34 cm observed in the control group. These measurements reflect considerable dilation of the left ventricle. The E-point to septal separation (EPSS), a minor indicator of left ventricular systolic function, varied between 0.35 and 1.40 cm in the dogs with DCM, while the mean EPSS in healthy dogs was substantially lower (0.14 cm), suggesting preserved contractility in the absence of myocardial dysfunction. The evaluation of ejection fraction (EF) and fractional shortening (FS) further confirmed systolic impairment in the DCM cohort. The EF values (with the Teichholz method) in affected dogs ranged from 53% to 76% (mean: 67%), and FS values ranged from 22% to 38% (mean: 32.6%). By contrast, control dogs exhibited higher EF (mean: 82%) and FS (mean: 50%) values, indicative of normal systolic performance. The left atrial-to-aortic-root ratio (LA/Ao), a sensitive marker of left atrial enlargement, was elevated in most DCM cases, ranging from 1.30 to 2.23, compared to a mean value of 1.40 in the control group. Notably, cases 1 and 6 displayed significant atrial dilation, with LA/Ao ratios exceeding 2.0, indicating severe hemodynamic compromise. The ventricular sphericity indices of the DCM dogs ranged from 1.16 to 1.73, reflecting the varying degrees of ventricular geometric remodeling and loss of ellipsoid shape. By contrast, the control group exhibited more preserved ventricular architecture, with a mean sphericity index of 2.29. Regarding arrhythmias, only one dog (Case 2) exhibited bradycardia; all other DCM cases and the control dogs showed no arrhythmic events following electrocardiographic examination (Table 3). In patients diagnosed with DCM, the LVIDDn values ranged from 1.90 to 2.59, while the LVIDSn values ranged from 1.19 to 1.46.

The systolic, diastolic and mean arterial pressure values are presented in Table 4. In the DCM group, the systolic arterial pressure (SAP) was 145.5 ± 22.56 mmHg (range: 111–181 mmHg), the diastolic arterial pressure (DAP) was 86.7 ± 25.09 mmHg (range: 50–119 mmHg) and the mean arterial pressure (MAP) was 105.8 ± 23.84 mmHg (range: 70–140 mmHg). In the healthy group, SAP was 131.5 ± 11.96 mmHg (range: 108–152 mmHg), DAP was 92.2 ± 14.71 mmHg (range: 72–120 mmHg) and MAP was 99.4 ± 16.12 mmHg (range: 72–129 mmHg). The statistical analysis revealed no significant differences between the healthy and DCM groups for any of the measured parameters (*p* > 0.05).

**Figure 1:**
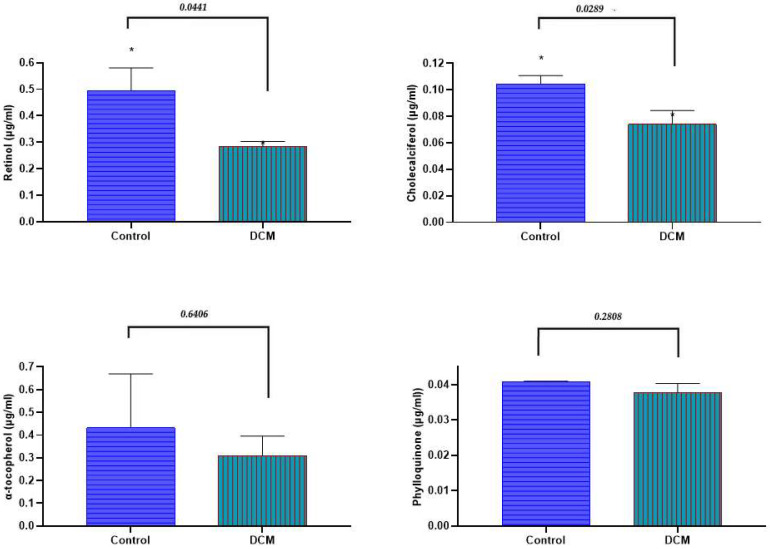
The bar chart shows the mean levels of retinol, cholecalciferol, α-tocopherol and phylloquinone in dogs with DCM and control. *: *p* < 0.05.

The mean plasma retinol concentration was 0.0285 ± 0.0018 μg/mL in the DCM group and 0.0495 ± 0.0085 μg/mL in the control group. The respective mean cholecalciferol concentrations were 0.0739 ± 0.0010 μg/mL and 0.1045 ± 0.0062 μg/mL. Retinol and cholecalciferol levels were both significantly lower in the DCM group than in the control group (*p* < 0.05). The mean plasma α-tocopherol concentrations were 0.3103 ± 0.0084 μg/mL and 0.4322 ± 0.235 μg/mL in the DCM and control groups, respectively, and the mean plasma phylloquinone concentrations were 0.0378 ± 0.0024 μg/mL and 0.0408 ± 0.0001 μg/mL, respectively. However, no statistically significant differences were observed between the groups for α-tocopherol or phylloquinone levels (*p* > 0.05) (see Figure 1).

## 4. Discussion

Currently, in dogs, NT-proBNP and high-sensitivity cardiac Troponin I are recognized as important biomarkers with moderate-to-high accuracy in distinguishing dogs with early structural or electrical cardiac abnormalities. Although neither biomarker plays a definitive role in diagnosis, both are particularly useful as screening tests for identifying at-risk dogs [28]. In addition, various studies have reported proteomic analyses aimed at identifying novel diagnostic biomarkers for idiopathic DCM in dogs [29,30,31]. The findings of this study, by revealing the relationship between DCM and fat-soluble vitamin levels, may contribute to the identification of therapeutic targets in the future and to the evaluation of the indication for vitamin supplementation in the treatment of the disease.

Retinol serves as a prohormone for tretinoin, also known as retinoic acid (RA), all-trans retinoic acid (ATRA) or vitamin A acid, and has been identified as a crucial vitamin form for early cardiac development [32,33,34,35]. During embryogenesis, RA plays an essential role in the specification and morphogenesis of cardiac structures through tightly regulated spatial and temporal expression patterns. Although the precise mechanisms remain to be fully elucidated, retinal dehydrogenase (RADH)—the enzyme catalyzing the oxidative conversion of retinol to ATRA—has been proposed as a key regulator of retinoid signaling during cardiogenesis [32]. In vitro studies have demonstrated that RA enhances the expression of cardiac-specific transcription factors such as GATA4, NKX2.5 and MEF2C and promotes cardiomyocyte formation and differentiation during fetal development [20,36]. In vivo evidence further supports the essential role of RA signaling in cardiomyocyte repair and regeneration following myocardial injury, as shown in murine models of infarction [37]. Collectively, these RA–cardiomyocyte interactions suggest a potential involvement of retinol and its active metabolites in the etiopathogenesis of cardiomyopathies.

Despite apparently adequate systemic retinol levels, myocardial ATRA concentrations have been reported to decline in both human and experimental models of heart failure [38]. This suggests a potential functional deficiency at the tissue level. In human studies, it has been reported that myocardial ATRA concentrations decrease in heart failure, and that this decline occurs independently of serum levels, underscoring the importance of tissue-level concentrations [39]. Similarly, another study reported a positive correlation between serum retinyl ester concentrations and hepatic vitamin A stores [40]. Therefore, although a relationship exists between tissue and serum retinoid values, it should be considered that serum concentrations may not always accurately reflect tissue levels. Local disturbances in retinoid metabolism or signaling pathways, such as decreased RALDH activity, downregulation of RA receptors or altered cellular retinol-binding proteins (CRBPs), could explain discrepancies between circulating and tissue-specific levels [41]. In the present study, all cases of dilated cardiomyopathy (DCM) were classified as idiopathic, as no other underlying etiologies were identified through diagnostic evaluations. Histopathological alterations, including cardiomyocyte degeneration, infiltration, deposition and atrophy, are known to contribute to cardiac dysfunction in predisposed canine breeds. While genetic mutations and familial forms of DCM have been identified, particularly in breeds such as Doberman Pinschers and Boxers, the majority of clinical cases remain idiopathic [5,42].

In view of the proposed link between grain-free, legume-rich diets lacking in taurine and L-carnitine and nutritionally induced DCM, all the dogs in this study were fed a balanced, commercially available adult maintenance diet to minimize nutritional variability. However, variations in dietary retinol content may still occur among different formulations, particularly in diets with low levels of animal-based ingredients. Previously reported plasma retinol concentrations in healthy dogs ranged from 0.34–0.52 μg/mL [43] to 0.71–1.14 μg/mL [44]. Freeman et al. [45] reported mean plasma retinol levels of 0.9 μg/mL (range: 0.8–3.0 μg/mL) in dogs diagnosed with DCM, with no statistically significant difference from healthy controls. However, in contrast to these earlier findings, the serum retinol concentrations observed in our DCM cohort were markedly lower than those reported in the literature. This observation supports the hypothesis that disruptions in retinoid metabolism may contribute to the pathogenesis of DCM. Furthermore, the decline in ATRA levels previously noted in heart failure [38] may reflect increased metabolic demand or impaired retinoid signaling, which could affect systemic retinol homeostasis. These findings highlight the need for further research into the potential benefits of retinol supplementation or the pharmacological modulation of RA pathways, particularly in the advanced stages of DCM.

Cholecalciferol (vitamin D_3_) is a biologically active form of vitamin D that is known to play a role in anti-inflammatory processes. It has also been investigated for its potential to protect the heart in human medicine [46]. Studies have demonstrated an association between vitamin D deficiency and cardiac hypertrophy, inflammation and myocardial remodeling [47,48]. Furthermore, supplementation with vitamin D_3_ has been reported to reduce plasma renin concentrations in individuals with heart failure, highlighting its potential regulatory role in the renin–angiotensin system [16]. While the precise mechanisms through which cholecalciferol influences cardiomyocyte function are unclear, the presence of vitamin D receptors (VDRs) and activating hydroxylases in ventricular cardiomyocytes suggests a direct involvement in myocardial physiology. Recent evidence suggests that disruption of vitamin D signaling may contribute to adverse cardiac remodeling, whereas sufficient levels may exert protective effects by attenuating structural and functional myocardial deterioration [17]. In our study, serum cholecalciferol concentrations were the presence of vitamin D receptors (VDRs) and activating hydroxylases in ventricular cardiomyocytes suggests a direct involvement in myocardial physiology This significant reduction may reflect an impaired vitamin D status associated with DCM pathogenesis, suggesting a potential link between vitamin D deficiency and cardiomyocyte dysfunction in affected dogs. However, it should be noted that intestinal malabsorption or altered hepatic metabolism due to chronic illness may also contribute to reduced serum concentrations. While it is unclear whether hypovitaminosis D is a cause or consequence of myocardial disease, our findings highlight the need for further research into the role of vitamin D in canine DCM, including its potential diagnostic and therapeutic applications.

The present study found similar plasma concentrations of α-tocopherol (vitamin E) and phylloquinone (vitamin K_1_) in dogs with DCM and healthy controls, with no statistically significant differences (α-tocopherol: *p* = 0.6406; phylloquinone: *p* = 0.2808). α-Tocopherol is a major fat-soluble antioxidant that exerts anti-inflammatory effects on cardiac tissue by mitigating the damage caused by oxidative stress, which plays a central role in myocardial injury and remodeling [49]. Phylloquinone, the primary dietary form of vitamin K, is mainly derived from plants and is often added to commercial pet food. It participates in several critical physiological processes, including blood coagulation, vascular calcification and bone metabolism [50]. Data on the role of phylloquinone in cardiovascular disease, particularly heart failure and DCM, remain scarce in both veterinary and human medicine. However, it has been implicated in myocardial health due to its involvement in vitamin K-dependent proteins, such as matrix Gla-protein (MGP), which inhibit pathological calcification. While this study did not reveal any statistically significant findings, the slightly lower mean concentrations observed in the DCM group could suggest a subclinical trend, though this is not sufficient to support a direct pathogenic role. Future studies evaluating functional indicators such as undercarboxylated MGP (ucMGP) or total antioxidant capacity may provide more meaningful insights into the biological activity of these vitamins.

This study has several limitations that should be acknowledged. Firstly, the relatively small sample size may have limited the statistical power to detect subtle differences in vitamin concentrations, particularly for α-tocopherol and phylloquinone. Secondly, the cross-sectional design precludes establishing temporal or causal relationships between vitamin deficiencies and the development or progression of DCM. Thirdly, while all dogs were fed a commercially available balanced diet, variations in the nutrient composition and bioavailability of different formulations could not be fully controlled or quantified. Additionally, the assessment was limited to serum or plasma vitamin concentrations, failing to evaluate tissue-specific levels or the functional activity of related metabolic pathways such as retinoid receptor expression or vitamin K-dependent protein activity. One of the other limitations was the unavailability of Holter monitoring for prolonged ECG screening to rule out occult DCM. Finally, other potential confounding factors, such as gastrointestinal absorption efficiency, breed-specific metabolic differences and concurrent subclinical conditions, could not be entirely excluded. To validate and expand upon these findings, and to explore the clinical relevance of vitamin-targeted interventions in canine DCM, future studies incorporating longitudinal designs, larger sample sizes and functional biomarker assessments, along with histopathological analysis of myocardial tissue, are warranted.

## 5. Conclusions

The findings of this study may suggest that alterations in vitamin A and D metabolism could be associated with the development of idiopathic DCM in dogs. However, given the limited sample size, these observations should be interpreted with caution. Serum α-tocopherol and phylloquinone levels did not differ significantly between dogs with DCM and healthy dogs, indicating that these fat-soluble vitamins are unlikely to be primary contributors to DCM pathogenesis in this cohort. Nevertheless, the slight reductions observed warrant further investigation in larger populations.

## Figures and Tables

**Table 1:** Hemogram results of DCM and control groups.

	DCM (*n* = 6)	Control (*n* = 10)	*p*
	Mean ± SD (Min–Max)	Mean ± SD (Min–Max)	
**RBC (M/µL)**	6.07 ± 1.22 (4.36–7.99)	7.15 ± 0.33 (6.38–7.75)	0.042 ^1^
**HCT (%)**	36.96 ± 6.74 (25.9–43.7)	47.45 ± 2.33 (44.4–53.3)	<0.001 ^1^
**Hgb (g/dL)**	13.93 ± 2.39 (9.9–16)	17.77 ± 0.95 (15.8–19.8)	<0.001 ^1^
**MCV (fL)**	61.11 ± 3.74 (54.8–64.9)	66.40 ± 1.95 (63–69.6)	0.002 ^2^
**MCH (pg)**	23.05 ± 3.74 (19.6–25.4)	24.85 ± 1.46 (21.8–27.8)	0.056 ^2^
**MCHC (g/dL)**	37.73 ± 1.12 (35.9–39.2)	37.42 ± 1.37 (34.6–49)	0.645 ^2^
**RDW (%)**	14.91 ± 0.57 (14–15.2)	12.92 ± 0.51 (12–14.1)	<0.001 ^1^
**RET (%)**	0.30 ± 0.10 (0.2–0.4)	N/A	
**RETIC (K/µL)**	16.66 ± 7.09 (8.7–22.3)	N/A	
**WBCs (K/µL)**	11.97 ± 4.18 (6.95–17.92)	9.46 ± 0.93 (7.26–11.1)	0.428 ^1^
**%NEU**	71.33 ± 4.37 (66.24–76.07)	72.06 ± 1.74 (69.45–75.91)	0.712 ^2^
**%LYM**	17.61 ± 3.69 (12.99–21.1)	18.81 ± 1.94 (16.16–22.26)	0.489 ^2^
**%MONO**	8.1 ± 3.41 (2.22–11.7)	6.51 ± 1.34 (3.94–8.75)	0.317 ^2^
**%EOS**	2.77 ± 2.00 (0.4–6.33)	2.34 ± 0.73 (0.52–3.40)	0.914 ^1^
**%BASO**	0.17 ± 0.10 (0.1–0.32)	0.26 ± 0.12 (0.14–0.59)	0.220 ^1^
**NEU (K/µL)**	8.55 ± 3.02 (4.6–12.52)	6.78 ± 0.55 (5.51–7.78)	0.073 ^1^
**LYM (K/µL)**	2.07 ± 0.74 (1.2–3.21)	1.80 ± 0.33 (1.17–2.47)	0.434 ^2^
**MONO (K/µL)**	1.05 ± 0.71 (0.21–1.6)	0.62 ± 0.14 (0.28–0.84)	0.313 ^1^
**EOS (K/µL)**	0.27 ± 0.12 (0.08–0.43)	0.21 ± 0.06 (0.05–0.28)	0.209 ^2^
**BASO (K/µL)**	0.015 ± 0.08 (0.01–0.03)	0.05 ± 0.04 (0.01–0.16)	0.016 ^1^
**PLT (K/µL)**	313.1 ± 79.1 (235–464)	274.2 ± 74.9 (118–435)	0.181 ^1^
**MPV (fL)**	9.76 ± 2.41 (7–12.8)	8.55 ± 0.46 (7.6–9.3)	0.513 ^1^
**PCT (%)**	10.7 ± 0.07 (0.34–0.49)	N/A	

^1^ Significance level of the Mann–Whitney U test for group comparisons. ^2^ Significance level of the independent samples *t*-test for group comparisons. RBCs = red blood cells; HCT = hematocrit; Hgb = hemoglobin; MCV = mean corpuscular volume; MCH = mean corpuscular hemoglobin; MCHC = mean corpuscular hemoglobin concentration; RDW = red cell distribution width; RET = reticulocytes; RETIC = reticulocytes; WBC = white blood cells; NEU = neutrophils; LYM = lymphocytes; MONO = monocytes; EOS = eosinophils; BASO = basophils; PLT = platelet count; MPV = mean platelet volume; PCT = plateletcrit; N/A = not available.

**Table 2:** Serum biochemistry results of DCM and control groups.

	DCM (*n* = 6)	Control (*n* = 10)	*p*
	Mean (Min–Max)	Mean (Min–Max)	
**GLU (mg/dL)**	95.83 ± 7.98 (85–108)	99.70 ± 3.83 (98–106)	0.208 ^2^
**Crea (mg/dL)**	0.77 ± 0.33 (0.48–1.2)	0.87 ± 0.20 (0.6–1.17)	0.476 ^2^
**BUN (g/dL)**	15.7 ± 3.99 (9–19.8)	18.27 ± 1.71 (16.7–22.6)	0.313 ^1^
**BUN/CREA**	22.9 ± 10.7 (14–41.2)	22.7 ± 4.95 (14.2–30.1)	0.882 ^2^
**PHOS (mg/dL)**	3.86 ± 0.76 (3–4.7)	3.59 ± 0.29 (3–3.8)	0.302 ^2^
**CA (mg/dL)**	10.6 ± 0.15 (10.4–10.7)	10.5 ± 0.14 (10.2–10.7)	0.313 ^1^
**TP (g/dL)**	7.10 ± 0.67 (6.3–8.1)	7.12 ± 0.35 (6.7–7.8)	0.938 ^2^
**ALB (g/dL)**	3.13 ± 0.53 (2.7–3.9)	3.38 ± 0.25 (3.2–3.7)	0.259 ^2^
**GLOB**	3.95 ± 0.97 (2.8–5.4)	3.73 ± 0.26 (3.2–4.1)	0.562 ^2^
**ALB/GLOB**	0.66 ± 0.22 (0.36–1)	0.87 ± 0.07 (0.86–1.09)	0.022 ^1^
**ALT (U/L)**	39.8 ± 10.2 (31–59)	51.4 ± 11.9 (39–69)	0.278 ^2^
**ALKP (U/L)**	62.6 ± 36.7 (26–121)	35.6 ± 8.34 (24–46)	0.181 ^1^
**GGT (mg/dL)**	10 ± 3.57 (6–14)	10.2 ± 1.19 (9–12)	0.949 ^2^
**TBIL (mg/dL)**	0.73 ± 0.54 (0.2–1.4)	0.40 ± 0.12 (0.2–0.5)	0.220 ^1^
**TCHOL (mg/dL)**	203.6 ± 74.4 (106–255)	270.9 ± 8.11 (265–283)	<0.001 ^1^
**AMY (U/L)**	979.6 ± 364.8 (545–1354)	788.4 ± 74.8 (604–840)	0.277 ^1^
**LIPA (U/L)**	168 ± 119.8 (32–300)	116.5 ± 90.5 (25–250)	0.274 ^1^

^1^ Significance level of the Mann–Whitney U test for group comparisons. ^2^ Significance level of the independent samples *t*-test for group comparisons. GLU = glucose; Crea = creatinine; BUN = blood urea nitrogen; PHOS = phosphorus; Ca = calcium; TP = total protein; ALB = albumin; ALT = alanine aminotransferase; ALKP = alkaline phosphatase; GGT = gamma glutamyl transferase; TBIL = total bilirubin; TCHOL = total cholesterol; AMY = amylase; LIPA = lipase.

**Table 3:** Echocardiographic variables of dogs with DCM and healthy groups.

	Groups	Mean	Std. Deviation	Min–Max	*p*
LVIDd (cm)	DCM	5.58	1.26	3.95–7.83	<0.001 ^1^
	Healthy	2.64	0.83	1.28–7.83	
LVIDs (cm)	DCM	3.68	0.59	3.07–4.78	<0.001 ^1^
	Healthy	1.34	0.52	0.47–2.31	
EPSS (cm)	DCM	0.83	0.37	0.35–1.40	<0.001 ^2^
	Healthy	0.14	0.05	0.07–0.20	
EF (%)	DCM	67.17	8.57	53–76	0.006 ^1^
	Healthy	81.60	8.69	68–96	
FS (%)	DCM	32.67	6.56	22–38	0.002 ^1^
	Healthy	49.70	9.76	36–70	
LA/Ao	DCM	1.84	0.32	1.30–2.23	0.002 ^1^
	Healthy	1.40	0.14	1.16–1.65	
Sphericity	DCM	1.42	0.27	1.16–1.73	0.004 ^1^
	Healthy	2.29	0.54	1.65–3.27	

^1^ Significance level of the independent samples *t*-test for group comparisons. ^2^ Significance level of the Mann–Whitney U test for group comparisons. LVIDd = left ventricular internal diameter at end diastole; LVIDs = left ventricular internal diameter at end systole; EPSS = E-point septal separation; EF = ejection fraction; LA/Ao = left atrium/aorta.

**Table 4:** Systolic, diastolic and mean arterial pressure values of DCM and healthy groups.

		Mean	Std. Deviation	Minimum	Maximum	*p* ^1^
SAP	DCM	145.5	22.56	111	181	0.124
	Healthy	131.5	11.96	108	152	
DAP	DCM	86.7	25.09	50	119	0.583
	Healthy	92.2	14.71	72	120	
MAP	DCM	105.8	23.84	70	140	0.528
	Healthy	99.4	16.12	72	129	

^1^ Significance level of the independent samples *t*-test for group comparisons. SAP: systolic arterial pressure, DAP: diastolic arterial pressure, MAP: mean arterial pressure.

## Data Availability

No new data were created or analyzed in this study. Data sharing is not applicable to this article.
